# Therapeutic Applications of Cannabinoids in Cardiomyopathy and Heart Failure

**DOI:** 10.1155/2020/4587024

**Published:** 2020-10-27

**Authors:** J. A. Garza-Cervantes, M. Ramos-González, O. Lozano, C. Jerjes-Sánchez, G. García-Rivas

**Affiliations:** ^1^Tecnologico de Monterrey, Escuela de Medicina y Ciencias de la Salud, Cátedra de Cardiología y Medicina Vascular, Monterrey, Nuevo León, Mexico; ^2^Tecnologico de Monterrey, Hospital Zambrano Hellion, TecSalud, Centro de Investigación Biomédica. San Pedro Garza García, Nuevo León, Mexico; ^3^Tecnologico de Monterrey, Hospital Zambrano Hellion, TecSalud, Centro de Medicina Funcional, San Pedro Garza García, Nuevo León, Mexico

## Abstract

A large number of cannabinoids have been discovered that could play a role in mitigating cardiac affections. However, none of them has been as widely studied as cannabidiol (CBD), most likely because, individually, the others offer only partial effects or can activate potential harmful pathways. In this regard, CBD has proven to be of great value as a cardioprotective agent since it is a potent antioxidant and anti-inflammatory molecule. Thus, we conducted a review to condensate the currently available knowledge on CBD as a therapy for different experimental models of cardiomyopathies and heart failure to detect the molecular pathways involved in cardiac protection. CBD therapy can greatly limit the production of oxygen/nitrogen reactive species, thereby limiting cellular damage, protecting mitochondria, avoiding caspase activation, and regulating ionic homeostasis. Hence, it can affect myocardial contraction by restricting the activation of inflammatory pathways and cytokine secretion, lowering tissular infiltration by immune cells, and reducing the area of infarct and fibrosis formation. These effects are mediated by the activation or inhibition of different receptors and target molecules of the endocannabinoid system. In the final part of this review, we explore the current state of CBD in clinical trials as a treatment for cardiovascular diseases and provide evidence of its potential benefits in humans.

## 1. Introduction

**Figure 1:**
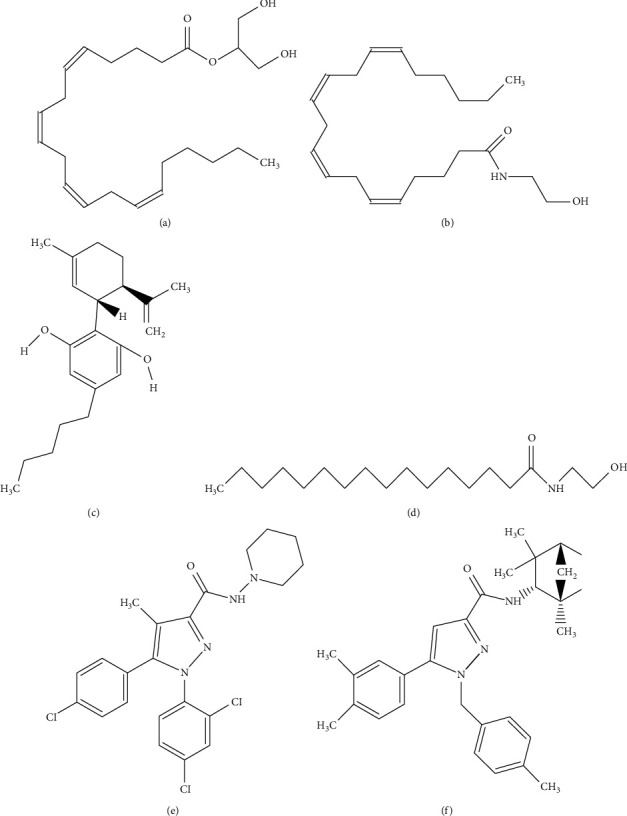
Molecular structure of cannabinoids of cardiovascular interest. (a) 2-arachidonoylglycerol, (b) anandamide, (c) CBD, (d) palmitoylethanolamide, (e) SR141716A, and (f) SR144528.

Cannabidiol (CBD) is one of the 113 identified phytocannabinoids found in *Cannabis sativa*. It is nonpsychotropic and constitutes up to 40% of the plant extract [1]. It can also be obtained synthetically. The molecular structure of CBD, and other cannabinoids, is shown in Figure 1. Since the identification of the cannabinoid receptor in the rat brain [2], there has been increasing interest among researchers in the use of cannabinoids to treat diseases. Of particular interest has been CBD, which, in addition to its lack of psychotropic activity, is a potent anti-inflammatory molecules, and reduces the production of reactive oxygen and nitrogen species (ROS/RNS), thus resulting in reduced tissue injury [3, 4]. Such effects can be taken advantage of in varied diseases, such as cardiovascular and neurodegenerative diseases, cancer, pain, obesity, and metabolic syndrome. For this range of applications, it is worth noting that CBD is only approved in 27 countries to treat multiple sclerosis. In the United States (US), the Food and Drug Administration (FDA) has approved its use in the treatment of Dravet and Lennox-Gastaut syndromes [5]. Even if it has been shown that CBD has effects on cannabinoid type 2 receptors (CB2s) in the brain [6] but not cannabinoid type 1 receptors (CB1s), there is still controversy regarding the specific pathways involved in its anti-inflammatory properties. Regarding the very production of CBD, the synthetic route is preferred over extracts for several reasons, including high composition purity, pesticide removal, consistent quality control between batches, and ease of scalability without using *Cannabis sativa* as the initial source. Moreover, some synthetics, such as 8,9-dihydrocannibidiol (H2CBD), do not activate the THC pathway, unlike CBD extracts [7, 8].

Cardiovascular diseases (CVDs) are the leading cause of mortality worldwide [9]. They have a set of characteristic molecular components, such as a proinflammatory profile present in heart failure (HF) [10], diabetic cardiomyopathy [11], and autoimmune myocarditis [5]. In addition, alterations to oxidative phosphorylation and mitochondrial reactive oxygen species (ROS) are linked to hypoxia [12] and ischemia/reperfusion injury [13, 14], where excess ROS and alterations to the renin-angiotensin-aldosterone system are linked to hypertension [15, 16]. Similarly, the handling of intracellular Ca^2+^ is paramount in HF as well as arrhythmias [17]. All these molecular components, associated with diverse CVDs, can be modulated with CBD administration. The objective of this study is to review the current evidence related to CBD and other nonpsychoactive cannabinoids, including their use in CVDs, especially HF and cardiomyopathies. From this evidence, we finalize with a proposed roadmap of the pathways of CBD's effects on CVDs.

## 2. Cardiovascular Effects of CBD

### 2.1. CBD as a Therapeutic Approach in Models of Cardiopathy

Many authors have studied the cardiovascular effects of CBD to explore its therapeutic potential in a variety of cardiomyopathies, from healing injury caused by several cell stressors to decreasing the incidence of cardiac damage. These effects are reported using different models: cells, tissues, and animals.  Table 1 summarizes the use of CBD in cardiomyopathies.

### 2.2. Cardiovascular Effects of CBD Observed In Vitro

The alteration of cardiovascular cells could compromise heart function, and vascular diseases are linked to proliferative and inflammatory responses. For example, Schwartz et al. [18] demonstrated that CBD inhibits the growth factor-mediated proliferation and migration of vascular smooth muscle cells (VSMC). Stanley et al. [19] characterized CBD-induced intracellular signaling using human aortic endothelial cells (HAECs), increasing vasorelaxation, and a decrease of proinflammatory proteins. Using HCAECs (coronary artery endothelial cells), Rajesh et al. [20] found that CBD attenuates the inflammatory response caused by high glucose concentrations in endothelial cells. Furthermore, CBD caused a reduction in oxidative and nitrosative stress in an in vitro study using primary human cardiomyocytes [11] by decreasing reactive oxygen/nitrogen species and NF-*κβ* expression. Similarly, using Chinese hamster ovary (CHO) cells transfected with a human Na_v_1.5 *α*-subunit and then submitted to high glucose conditions, Fouda et al. [21] found that CBD mitigated the elevation of ROS and increased the length of action potential provoked by these conditions, suggesting that the antioxidant property of CBD, in conjunction with a sodium channel inhibitor, may have cardioprotective effects against arrhythmia and cytotoxicity caused by high glucose environments.

Ali et al. [22] observed the effects of CBD on the contractility and electrophysiological properties of rat ventricular myocytes. The results indicate that the inhibition of Ca^2+^ signaling underlies the negative inotropic effects of CBD in myocytes, presenting a reduction in maximal shortening amplitudes with no alteration of the time course of contraction. However, Robertson-Gray et al. [23] observed a cardioprotective effect of CBD against l-*α*-lysophosphatidylinositol (LPI), an endogenous ligand of G protein-coupled receptor 55 (GPR55), a putative cannabinoid receptor, which is also elevated in patients with the coronary syndrome. The injury was decreased by CBD acting as an antagonist of the GPR55 receptor. A similar effect of GPR55 antagonism was observed by Marichal-Cancino et al. [24], as CBD blocked the LPI-induced inhibition of vasopressor responses produced by noradrenaline, suggesting this receptor may play a role in CBD's effects.

### 2.3. Cardiovascular Effects of CBD Observed Ex Vivo

During an ex vivo experiment performed by Stanley et al. [25], improved myocardial function was observed due to CBD-enhanced acetylcholine-induced vasorelaxation in Zucker diabetic rat aorta. Similarly, Wheal et al. [26] reported the improvement of endothelium-dependent vasorelaxation in mesenteric arteries ex vivo in diabetic rats. After seven days of treatment with CBD, vasorelaxation was mediated by COX and NO mechanisms, given that indomethacin or L-NAME inhibited the effect. Furthermore, these vasorelaxant effects observed in diabetic rats were not observed in the control groups, suggesting that CBD reveals its positive effect when vascular dysfunction is present. In a study conducted by Stanley et al. [19], CBD caused significant vasorelaxation of precontracted human mesenteric arteries compared in the control group, and the effect was significantly reduced by the endothelium removal and the use of a high potassium physiological salt solution. The use of AM251 and LY320135, CB_1_ receptor antagonists, and the transient receptor potential cation channel (TRP) desensitizer capsaicin abolished the vasorelaxation effect of CBD. Meanwhile, O'Sullivan et al. [27] studied the functional vascular effect of CBD in rat aortae. CBD caused vasorelaxation of the precontracted aorta in a time-dependent manner. In contrast, in this study, pretreatment with AM251, AM630, or capsaicin did not affect the vascular response to CBD, suggesting neither CB_1_, CB_2_ receptors, nor TRP in aorta, respectively, was involved.

### 2.4. Cardiovascular Effects of CBD Observed In Vivo

#### 2.4.1. Use of CBD on Hypertension Models

Cannabinoids are known to reduce oxidative metabolism in different tissues, including the heart [28]. Using a primary and secondary hypertension rat model, Remiszewski et al. [29] found a reduction in oxidative stress in both the heart and plasma when rats were treated with CBD but, unexpectedly, did not modify the blood pressure (BP) or heart rate (HR). In another hypertensive model, Kossakowski et al. [30] observed the cardiovascular effect of CBD on conscious and anesthetized, spontaneously hypertensive rats. The intravenous (i.v.) administration of CBD to anesthetized rats caused a dose-dependent decrease in HR, as well as systolic and diastolic blood pressure, more markedly in hypertensive rats than normotensive rats. However, the study reported that in conscious rats, the effect was not observed due to a slowly infused intraperitoneal (i.p.) administration of CBD.

#### 2.4.2. Use of CBD on Ischemia/Reperfusion and Arrhythmias

The cardioprotective effect of CBD has been studied in different cardiomyopathies, including ischemia/reperfusion (I-R) and arrhythmias. Durst et al. [31] studied the protection provided by CBD after I-R injury. Decreased infarct size was observed when Sprague-Dawley rats were treated with CBD, while the area at risk was similar to the control group. There were no differences in myocardial contractility or remodeling parameters. Furthermore, the inflammatory response was lower in CBD-treated hearts than in the I-R group, including decreased levels of IL-6, suggesting that the cardioprotective effect of CBD may be caused by a systemic immunomodulatory effect. Feng et al. [32] observed the facilitated restoration of the left ventricular function when rabbits were administered with CBD. Walsh et al. [33] tested the effect of CBD on cardiac arrhythmias and the infarct size when administered immediately before I-R on Sprague-Dawley rats. Administering CBD caused a reduction in the infarct size and collagen-induced platelet aggregation, as well as significantly reduced the number of ischemia-induced ventricular arrhythmias compared to the vehicle-treated group. Similarly, using an in vivo I-R model, Gonca et al. [34] observed a decreased incidence and duration of ventricular tachycardia on Wistar rats. These studies show that CBD may be beneficial in the prevention of arrhythmias caused by different conditions, such as heart failure.

#### 2.4.3. Use of CBD on Cardiomyopathies with Concurrent Diseases

The clinical outcomes associated with heart failure are considerably worse for patients with concurrent diseases, such as immunological pathologies or diabetes mellitus. Therefore, CBD represents a potential treatment for cardiovascular complications, including diabetic cardiomyopathy, which is characterized by myocardial left ventricular dysfunction. Wheal et al. [35] found that CBD improved vasorelaxation in diabetic rats, as there was a 40% increase in vasorelaxation in diabetic rats when compared to lean rats. Meanwhile, Rajesh et al. [11] found during diabetic cardiomyopathy that attenuated cardiac fibrosis and oxidative/nitrosative stress improve myocardial function. In a model of autoimmune myocarditis, Lee et al. [5] observed that CBD provides inflammatory and injury protection. Administration of CBD to immunocompromised mice caused a decrease in T-cell markers as well as monocyte cell activators. These CBD-treated mice showed a lower CD3^+^ and CD4^+^ T-cell-mediated inflammatory response and decreased myocardial injury and fibrosis.

CBD may play an important role in cardioprotection when heart injury is caused by the treatment of another pathology, like doxorubicin, an anticancer antibiotic known for its cardiotoxicity. Regarding, Fouad et al. [36] reported in vivo CBD cardioprotection. In the study, CBD caused a decrease in several heart injury markers elevated by doxorubicin, including serum creatine kinase-MB and cardiac troponin T (cTnT), and cardiac malondialdehyde attenuated inflammation and oxidative stress. Similarly, Hao et al. [37], using an in vivo doxorubicin-induced cardiomyopathy model, observed attenuated cardiac oxidative and nitrative stress and improved mitochondrial function and biogenesis which may contribute to its beneficial properties in tissue injury.

#### 2.4.4. Use of CBD on Cardiac Stress Conditions

Different kinds of stress may stimulate the sympathetic nerve activity affecting systemic metabolism. Resstel et al. [38, 39] and Gomes et al. [40] studied the effect of CBD on cardiovascular responses induced by restraining movement. Stress caused an increase in the animals' mean arterial pressure and heart rate. Pretreatment with CBD did not affect the baseline of the cardiovascular parameters, but it attenuated the increase in HR and mean arterial pressure (MAP) in stressed animals. CBD caused a reduction of pressure and tachycardic responses in a dose-dependent manner. Similarly, Granjeiro et al. [41] observed attenuation of the increase in MAP and HR after restraint stress in rats administered CBD. The results in these studies indicate there were no changes in the baselines of cardiovascular parameters within the control group, suggesting that CBD may act specifically on stress-related cardiovascular pathways.

### 2.5. Proposed Mechanisms and Molecular Pathways of CBD from Different Cardiovascular Models

**Figure 2:**
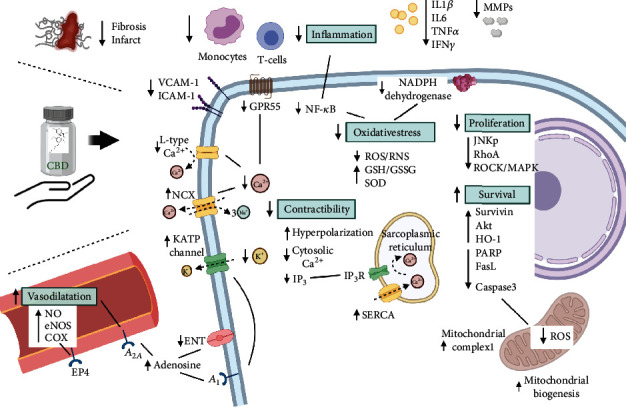
Mechanisms and molecular pathways of the CBD administration in the cardiovascular system. CBD is involved in regulating several pathways, protecting cardiomyocytes from inflammation and oxidative stress, regulating the Ca^2+^/K^+^ intake, decreasing immune proliferation, and promoting cellular survival. CBD helps to protect mitochondria and regulates their biogenesis, improving the cellular energy supply. In vascular vessels, CBD causes vasodilatation, lowering blood pressure, and protecting the heart. Arrows indicate changes in activity for each molecule/mechanism/inflammatory cell.

Based on the collected evidence in the studied models pointing to CBD as a promising cardioprotective therapy, we performed a thorough analysis of the pleiotropic mechanisms involved, as well as molecular pathways and target molecules that are, to some extent, affected by the CBD administration. We identified five main mechanisms modulated by CBD that are responsible for the beneficial effects observed in cardiac dysfunction and heart failure: (a) oxidative and nitrosative stress, (b) the inflammatory state, (c) effect on vasorelaxation, (d) the regulation of cardiac contractibility, and (e) antiproliferative and antiapoptotic properties. All of these mechanisms are explained in greater detail below and summarized in Figure 2.

#### 2.5.1. Oxidative and Nitrosative Stress

CBD is well known as a potent antioxidant due to its capacity to modulate the production of reactive oxygen and nitrogen species (ROS/RNS). Rajesh et al. [11] observed that the production of superoxide anion radicals was diminished by CBD treatment as a result of the reduced mRNA expression of the NADPH oxidase subunits p22phox, p67phox, and gp91phox, which are components of the oxidase system. CBD also reduces superoxide anion by increasing SOD and the glutathione peroxidase activity [1, 2], elevating the GSH/GSSG ratio, and reducing lipid peroxidation and protein carbonyl groups formation [11, 36, 37]. CBD redox protection is not limited to its antioxidant properties modulating ROS production and degradation. It also has a significant impact on reducing nitric oxide- (NO-) mediated stress by decreasing peroxynitrite and nitrotyrosine formation [36], limiting the generation of 4-hydroxynonenal (4-HNE), most likely by reducing lipid peroxidation, resulting in a marked reduction in the 3-nitrotyrosine (3-NT) generation [5, 11], which is indicative of a protective effect against RNS damage.

The role of mitochondria in cellular redox reactions is one of the most important contributions to electron transfer balance. Hao et al. [37] observed the cardiotoxicity derived from the use of doxorubicin which can be attenuated by CBD therapy, as it can activate mitochondrial complex 1 providing additional oxidative protection accompanied by an increase in the number of mitochondria and biogenesis stimulation. The mechanism that involves mitochondrial ROS production and biogenesis possibly depends on the mitochondrial Ca^2+^ uptake promoting the dephosphorylation of transcription factor A (TFAM), which consequently induces mitochondrial biogenesis [42].

#### 2.5.2. Inflammation

Most cardiac pathologies result in chronic inflammation, cellular damage, and subsequently, cell death and fibrotic tissue formation. In this context, CBD has been widely proven to be a major mediator of inflammation by reducing the expression of inflammatory cytokines [5, 11, 36], decreasing the production of adhesion molecules [11, 20] and matrix metallopeptidases (MMPs) [37] and consequently limiting the damage caused by the migration and infiltration of immune cells in the cardiac tissue. According to Fouad et al. [36], one of the most important targets of CBD in the inflammatory pathway is NF-*κβ*, restraining its phosphorylation and nuclear internalization, a mechanism most likely mediated via ROS reduction [19, 36, 37]. As a result of NF-*κβ* inhibition by CBD in the evaluated experimental models, there is a diminished expression of cytokines, lower infiltration by T-cells and neutrophils, and reduced IFN-*γ* [43] and myeloperoxidase secretion by these cells [32]. Thus, CBD limits damage to the extracellular matrix, decreases monocytic invasion, which can differentiate in myofibroblasts [31, 32], and decreases the production of collagen [11] and fibrosis formation, resulting in the maintenance of cardiac function. With lower collagen production, a decrease in collagen-induced platelet aggregation has been observed [33, 34], a condition exacerbated in ischemic conditions, dampening the risk of thrombotic formation.

In the animal models of myocardial infarction, the CBD administration improved the cardiovascular function, as evidenced by reductions in the infarct size and the area of fibrosis, as well as lower blood levels of IL-6 [31] and cardiac troponin cTnI, which are markers of tissular damage [32]. Furthermore, in chronic inflammatory diseases, tryptophan metabolism is affected, leading to a failure in the capacity of these patients to properly activate an immune response. CBD has a beneficial effect on these patients by reducing the indoleamine-2,3-dioxygenase (IDO) enzyme activity, which is responsible for converting tryptophan into kynurenine metabolites. The IDO enzyme activity is induced by IFN-*γ*, and the CBD administration prevents its activation [33, 43]. Tryptophan is also necessary for the synthesis of NAD^+^, which is an oxidizing agent, and serotonin, a neurotransmitter responsible for the antidepressant effect of cannabinoids, thus impacting the patient's mood and quality of life [33, 43].

#### 2.5.3. Vasorelaxation

Of the proposed mechanisms of CBD cardioprotection, vasorelaxation undoubtedly plays a major role in limiting damage to the cardiac tissue over time in the presence of hypertension, heart failure, and chronic vascular diseases. CBD stimulates the production of endothelial nitric oxide synthase (eNOS) [19] and hence, vasorelaxation via nitric oxide (NO) synthesis [36, 37].

Stanley et al. [19] stated that the vasorelaxant effect of CBD is mediated by activating CB1 receptors and transient receptor potential (TRP) channels, as the use of an antagonist for both inhibited vasorelaxation. In the same study, CBD also activated endothelial-bound cannabinoid receptor- (CBe-) mediated vasorelaxation, but it does not seem to be a direct interaction. Another strong candidate for a direct target is PPAR-*γ*. Although CBD has been proven to be a functional agonist of this receptor, it lacks the adverse side effects reported with the use of PPAR-*γ* agonists, namely, weight gain, fluid retention, and congestive heart failure. However, antagonizing this receptor only partially reduced the CBD vasorelaxant effect [27].

In arteries, the CBD enhanced COX activity, stimulating the production of prostaglandin E2, a potent smooth muscle vasodilator, activating EP4 receptors as described by Wheal et al. [26, 35]. In the endothelium, the COX activity may be mediated by the acetylcholine (ACh) via eNOS activation [26, 35]. CBD is known to suppress cellular adenosine intake, hence increasing its interstitial concentration and resulting in increased *A*_2_*a* receptor activation and elevated NO production in the smooth muscle, causing vasodilatation [36, 37]. Notably, iNOS' contribution to CBD vasorelaxation appears to be negligible since its expression is downregulated by CBD, most likely because iNOS is activated by NF-*κβ* and other pr-inflammatory stimuli, such as TNF-*α*, which are suppressed by CBD [11, 20, 37].

#### 2.5.4. Cardiac Contractility

The effects of endocannabinoids on the heart have been widely studied. They have protective functions in the cardiac tissue under stressful conditions, such as arrhythmias and myocardial infarction [44]. CBD has multiple interactions with ion channels and membrane receptors that alter the distribution of electrical charges inside the cell, modulating myocyte contractility. In Ca^2+^ homeostasis, CBD causes the inhibition of L-type Ca^2+^ channels, impeding the internalization of additional Ca^2+^ from the interstitial medium. When coupled with a reduction in the IP_3_-mediated release of Ca^2+^ from the sarcoplasmic reticulum, it results in difficulty depolarizing the cellular membrane, restraining the triggering of the myocyte action potential [23]. In pathological conditions involving high extracellular K^+^, CBD can modulate the activation of the Na^+^/Ca^2+^ exchanger (NCX) to release additional Ca^2+^ from the cytosol into the extracellular space [33]. Furthermore, by reducing the oxidative and inflammatory cascade, CBD can preserve the SERCA activity and promote Ca^2+^ storage in the sarcoplasmic reticulum [45]. Through these effects, the ionic balance is protected, and cytosolic overload is reduced [5] In this context, proarrhythmogenic events can be triggered by serum mediators of chronic inflammation modifying spontaneous Ca^2+^ release events, which provide a substrate for ventricular arrhythmias [46].

At the mitochondrial level, CBD has minor influence over mitochondrial Ca^2+^ regulation, as Walsh et al. [33] described a lack of CBD participation in the mitochondrial permeability transition. Interestingly, CBD can antagonize the activation of the GPR55 receptor [23], avoiding IP_3_-dependent Ca^2+^ liberation from the sarcoplasmic reticulum, limiting the entry of Ca^2+^ into the mitochondria during ischemia/reperfusion, and preventing further injury caused by the overflow of Ca^2+^. In this sense, avoiding mitochondrial Ca^2+^ overload has been established as a paradigm of cardioprotection in cardiac ischemia/reperfusion injury [47, 48].

CBD has a wide influence over the cellular membrane's capacity to regulate the ionic balance. CBD acts as a competitive inhibitor of the equilibrate nucleoside transporter (ENT), impeding the cellular uptake of adenosine, augmenting its extracellular concentration, and allowing the increased activation of adenosine *A*_1_ receptors [34, 39, 41], which, in turn, activates potassium K^+^-ATP channels and causes hyperpolarization of the cell membrane, producing an antiarrhythmic effect by decreasing cardiomyocyte excitability [19, 27, 33, 34]. Finally, CBD is also an agonist of 5-HT1A receptors, acting as an anxiolytic, possibly lowering the mean arterial blood pressure, producing sympathetic inhibition, and reducing ventricular arrhythmias [39, 41].

#### 2.5.5. Antiproliferative and Antiapoptotic Effects

Treatment with CBD can prevent cellular and tissular events, such as ischemia/reperfusion, and to some extent, reduce the infarct size. The mechanisms involved are a combination of the promotion of cellular survival, the inhibition of apoptosis, and extracellular matrix conservation by avoiding immune cellular expansion and migration. It has been noted that the enzyme HO-1 participates in protecting the cardiac and vascular tissue from aberrant cellular proliferation and migration. Schwartz et al. [18] demonstrated the role of CBD in upregulating the expression of HO-1, thus providing cardioprotection. In the presence of an inflammatory process, the HO-1 pathway activation by CBD could be of great importance to limit tissular damage from immune cell proliferation and invasion, as these cells' mitosis is activated by ROS via the induction of JNK phosphorylation [11, 19]. The CBD administration can also be beneficial to stressed cardiac cells by inducing the expression of survivin [36] and Akt phosphorylation [11, 19], both of which influence cell survival.

Under ischemia/reperfusion injury, CBD attenuates the RhoA activation [23] and its pathways ROCK/p38 and MAPK/MAPKAPK2 [11, 23], which are activated in response to inflammatory and proliferative stimuli. CBD plays a significant antiapoptotic role by protecting the cell from oxidative damage and Ca^2+^ overload, preventing the activation of caspase 3 cleavage, PARP activity, FasL expression, and chromatin fragmentation [11, 36, 37]. Both the antiproliferative and the antiapoptotic effects are naturally modulated by the action of endocannabinoids. CBD is known to inhibit the enzyme fatty acid amide hydrolase (FAAH), resulting in increased levels of available systemic endocannabinoids [35], which is an indirect mechanism of cellular protection.

### 2.6. Other Cannabinoids of Cardiac Significance

The endocannabinoid system has two distinct membrane receptors, CB_1_ and CB_2_, which are activated through endogenous cannabinoid ligands, as well as plant-derived cannabinoids and their synthetic analogs. However, some cannabinoids exhibit vasodilator effects via endothelial receptors distinct from CB_1_ and CB_2_ [49].  Table 2 summarizes the use of cannabinoids in different cardiomyopathies.

#### 2.6.1. Cardioprotective Effect of Cannabinoids against Ischemia/Reperfusion Injury

A variety of mechanisms of protection from cardiac ischemia-reperfusion injury implicate endocannabinoids. Ischemic preconditioning is well known for its potent cardioprotective effects, resulting in smaller infarct sizes, reduced risk of I-R arrhythmias, and improved recovery of ventricular function. Joyeux et al. [50] reported that isolated hearts preconditioned with heat stress reduced the infarct size significantly after an I-R sequence. This protection was abolished when treating isolated hearts with a CB_2_ receptor antagonist, SR144528, implicating the CB_2_ receptor in the observed cardioprotection.

Lépicier et al. [51] studied the protective effect of arachidinoylethilenamide (i.e., anandamide [ACEA]), 2-arachidonoylglycerol (2-AG), and palmitoylethanolamide (PEA) (agonists of CB_1_ and CB_2_ receptors) against I-R. They observed that treatment with PEA and 2-AG allowed the heart function recovery during reperfusion in terms of maximum dP/dt and left ventricular end-diastolic pressure (EDP). In presence of rimonabant (SR141716A), a selective CB_1_ antagonist, it still allowed the complete function recovery by PEA but cut the function recovery of 2-AG in half. However, the CB_2_ inverse agonist SR144528 allowed no recovery by PEA or 2-AG. They also observed that PEA and 2-AG decreased the levels of creatine kinase (CK) and lactate dehydrogenase (LDH), two biochemical markers of ischemic injury, as well as the infarct size. The decreased levels of CK and LDH were absent when treating hearts with anandamide. Using ACEA and JWH015, synthetic agonists of CB_1_ and CB_2_ receptors, respectively, activated these receptors and could protect the heart from ischemia. Also, using SB203580 and PD98059, inhibitors of p38 MAP kinase and the ERK1/2 pathway, respectively, demonstrated the participation of p83 and ERK1/2 in the cardioprotective effects of PEA, as the protection was abolished when using the inhibitors.

The contribution of endogenous cannabinoids in the protective effect of I-R on endothelial function was reported by Bouchard et al. [52]. They analyzed the vasodilatory response to serotonin (5-HT) and sodium nitroprusside (SNP). Hearts submitted to a preconditioning ischemia treatment preserved 5-HT vasodilatory effects. Meanwhile, the presence of either SR141716A or SR144528 abolished the 5-HT vasodilatory response, suggesting endogenous cannabinoids play a role in endothelial protection by acting on CB_1_ and CB_2_ receptors. This role was confirmed by perfusion with 2-AG, PEA, and anandamide following I-R. Perfusion with both 2-AG and PEA prevented an ischemia-induced reduction in vasodilation due to 5-HT, as they mimic the protection of preconditioning on the endothelial function. However, Underdown et al. [53] reported a significant reduction in the infarct size when using anandamide or methanandamide, its nonhydrolyzable analog. This protective effect was blocked when treated with either SR141716A or SR144528, CB_1_, and CB_2_ receptors antagonists, respectively. They suggest that the infarct-limiting action of anandamide requires costimulation of CB_1_ and CB_2_ receptors, as well as interaction with another cannabinoid receptor subtype, considering that the use of both ACPA or JWH-133, agonists of CB_1_ and CB_2_, respectively, did not mimic the effect of anandamide. Similarly, in anesthetized rats, Zakrzeska et al. [54] observed that anandamide and methanandamide caused a decrease in MBP without affecting HR, and these effects were reduced by CBD and O-1918, a GPR18 antagonist, suggesting the participation of other receptors, in addition to CB_1_/CB_2_ receptors.

Using the synthetic ligands of the CB_2_ receptor WIN55212-2 and JWH-133, Di Filippo et al. [55] and Montecucco et al. [56] demonstrated that the CB_2_ receptor activation confers protection against myocardial damage associated with I-R. The protective effect was abolished by AM630, a highly selective CB_2_ receptor antagonist. Furthermore, WIN55212-2 inhibited the local generation of leukocyte activators, cytokines, and chemokines, which are promoters of leukocyte-endothelium interaction, causing the inhibition of leukocyte-dependent damage of infracted myocardium [55], and JWH-133 reduced the production of superoxide species significantly and increased ERK1/2 and STAT-3 phosphorylation, pathways involved in cardioprotection [56].

Gorbunov et al. [57] reported the use of the CB_1_ and CB_2_ agonist HU-210 to reduce the infarction effect of reperfusion after local ischemia in the heart by mimicking the postconditioning phenomenon. In the study, HU-210 decreased the infarction size in the area at the risk ratio significantly, preventing reperfusion injury after ischemia. HU-210 decreased the pumping function of the heart, leading to a reduction of its work and oxygen demand during reperfusion, probably promoting cardiomyocyte survival during reperfusion. Moreover, HU-210 decreased end-diastolic pressure values during the reperfusion period, suggesting a reduction in the Ca^2+^ overload of cardiomyocytes.

#### 2.6.2. Cardioprotective Effect of Cannabinoids against Arrhythmias

Coronary occlusion and reperfusion cause arrhythmogenic effects in the myocardium, mostly modulated by a variety of neurotransmitters with the cardiovascular activity. Ugdyzhekova et al. [58] reported an increase of 50% in rats resistant to arrhythmogenic effects caused by epinephrine when treated with anandamide. The use of SR141716A and SR144528 did not affect the antiarrhythmic activity of anandamide, suggesting that the effect is mediated via anandamide receptors localized in the myocardium other than CB_1_ and CB_2_. They also suggest that the antiarrhythmic effect is caused by a decrease in the cAMP activity of L-type Ca^2+^ channel blockage in cardiomyocytes. Using the agonist HU-210, Ugdyzhekova et al. [59] reported an antiarrhythmic effect of the agonist by activating CB_2_ receptors and inhibiting cAMP synthesis, which acts as an arrhythmogenic factor. Using an I-R model, Krylatov et al. [60, 61] tested anandamide and methanandamide improvement in cardiac resistance to arrhythmias, observing that both cannabinoids possess the antiarrhythmic activity. Using L-NAME and glibenclamide as an inhibitor of NO synthase and K_ATP_ channels, respectively, no changes in the antiarrhythmic activity of both cannabinoids were observed. This suggests that the antiarrhythmic effect of endocannabinoids is not associated with the activity of NO synthase and K_ATP_ channels but is correlated with CB_2_ receptor stimulation.

#### 2.6.3. Hemodynamic Effect of Cannabinoids

Endocannabinoids play a crucial role in hemodynamic changes, a common complication of acute myocardial infarction (MI), and other cardiomyopathy complications. Wagner et al. [62] reported the contribution of activated vascular CB_1_ receptors to hypotension after experimental MI. Use of the CB_1_ antagonist SR141716A prevented hypotension after MI but aggravated early endothelial dysfunction and had a detrimental effect on early survival, similar to hemorrhagic shock. Endocannabinoid-induced vasodilation in cardiogenic shock also helps maintain adequate tissue perfusion in the face of decreased cardiac output and a compensatory increase in sympathetic vasoconstrictor tone. Matouk et al. [63, 64] reported a cardioprotective effect of abnormal cannabidiol (abn-CBD), a synthetic regioisomer of CBD, using in vivo diabetic cardiomyopathy models. Hemodynamic effects were studied by the chronic activation of the endocannabinoid receptor GPR18 in the absence or presence of O-1918, a GPR18 antagonist. Abn-CBD reduced blood pressure significantly with no effect on the heart rate, improved the left ventricular contractility index, and reduced LVEDP. The blockage of GPR18 with O-1918 abolished the effects obtained with abn-CBD. The observed hypotension caused by abn-CBD was associated with an increase in aortic endothelium-nitric oxide synthesis and in circulating NO and cGMP. Although abn-CBD did not influence hypertrophy or impaired glycemic control, it alleviated the diabetes-induced reduction in LV contractility and the relaxation index, LVEDP. These effects were observed due to the ability of abn-CBD to alleviate reductions in circulating adiponectin (ADN) and NO levels, increasing the myocardial GPR18 expression, reversing elevation in the cardiac AdipoR1 expression, as well as restoring myocardial Akt, ERK1/2, and eNOS phosphorylation, and restoring NO levels and redox status. GPR18 likely mediates cardioprotection because the concurrent administration of O-1918 with abn-CBD abolished the functional and biochemical responses [65, 66]. Similarly, virodhamine, a recently identified endocannabinoid, may act as an endothelium-dependent vasorelaxant compound via the abn-CBD receptor (GRP18) given that O-1918 inhibited the relaxation that virodhamine caused on isolated rat mesenteric arteries, as reported by Ho et al. [67], via activation of the Ca^2+^-activated K^+^ channel. However, Bondarenko et al. [68] reported that abn-CBD might mediate the vasodilation activity via the GPR-18-independent activation of high-conductance Ca^2+^-activated K^+^ channels (BK_Ca_) in mice aorta endothelial cells. Additionally, Ho et al. [69] determined that the relaxing effect of abn-CBD in the small mesenteric arteries of rats is caused by the endothelium-dependent K^+^ channel activation and Ca^2+^ channel inhibition. Baranowska-Kuczko et al. [70] observed the effect of anandamide in rat pulmonary arteries. Anandamide caused the endothelium-dependent vasorelaxation of precontracted pulmonary arteries with U-46619 by activating O-1918-sensitive endothelial cannabinoid receptors. In another study, Su et al. [71] observed the relaxation of permeabilized pulmonary arteries by 2-AG ether, a metabolically stable cannabinoid, and that abn-CBD is mediated by CB1 and abn-CBD receptors. This effect suggests the potential use of endocannabinoids, such as anandamide and 2-AG, in the treatment of pulmonary arterial hypertension. A similar endothelium-dependent vasorelaxation effect was observed by Milman et al. [72] in the isolated mesenteric arteries and abdominal aorta of rats caused by *N*-arachidonoyl-L-serine, an anandamide chemically related compound. During liver cirrhosis, heart functions are compromised, causing hyperdynamic circulation and leading to systemic hypotension and decreased peripheral resistance, termed cirrhotic cardiomyopathy. An in vivo model reported by Bátkai et al. [73] indicated an increased concentration of anandamide in the heart of cirrhotic rats, acting as an endogenous agonist of the contractile dysfunction associated with liver cirrhosis. The use of a CB_1_ antagonist, AM251, corrected the altered hemodynamic functions in cirrhotic rats, causing a gradual increase of MAP, but it caused no changes in normal rats. Cardiac hypertrophy, characterized by increased myocardial mass due to hemodynamic stress or cardiac injury, is considered a risk factor of heart failure. Using CB-13, a CB_1_/CB_2_ dual agonist, Lu et al. [74] decreased in vitro hypertrophy in the ventricular myocytes of rats by inhibiting myocyte enlargement and brain natriuretic peptide (BNP) expression and hypertrophy markers, as well as the activation of AMP-activated protein kinase (AMPK) and eNOS signaling. Their study suggests that a CB_1_/CB_2_ dual cannabinoid agonist with low brain penetration could achieve cardioprotection and avoid undesirable central nervous system CB_1_-mediated effects. It is notable that protective effects caused by the endocannabinoid system highly involve the CB_2_ receptor activation. It is possible that new natural or synthetic compounds acting as an agonist of this receptor exhibit cardioprotective effects, leading to a decrease in the infarcted size/area, leukocyte-dependent damage of the infarcted myocardium, inhibition of superoxide species production, Ca^2+^ overload, or activation of relevant protective pathways, such as p38, ERK1/2, and STAT-3, thus minimizing heart damage.

## 3. Bioavailability and Side Effects of CBD

### 3.1. Pharmacokinetic Parameters of the Oral CBD Administration in Healthy Humans

As CBD is typically administered orally or by inhalation in humans, knowing the pharmacokinetics of CBD is important to establish therapeutic doses and treatment regimens for patients with mild or severe illness conditions. Taylor et al. [75] studied the pharmacokinetics of an orally administered, highly purified CBD pharmaceutical formulation in healthy patients. The study evaluated the effect of single and multiple administrations, as well as the effect of food on pharmacokinetic parameters. Four different doses (1500, 3000, 4500, and 6000 mg/day) were administered in a single-dose trial. Maximal CBD concentration (Cmax) in plasma increased in a dose-dependent manner, but its time of maximal absorption (4–5 h) did not vary between the different doses. Similarly, the elimination half-time was similar for all doses (14–17 h). Two different doses, 300 and 1500 mg twice daily, were used in the multiple-dose trial. Maximal CBD concentrations were 1.6-fold higher than those in the single-dose trial, but time to maximal absorption was similar (3 h). Likewise, elimination half-times increased 4-fold when compared to the single-dose trial, suggesting a degree of time dependency in the elimination of CBD upon multiple dosing. In the multiple-dose trial, there were no significant differences in elimination half-times with CBD doses, indicating a decrease in bioavailability due to solubility-limited absorption at higher doses. During the food effect trial, a fed state increased the Cmax of CBD when compared to the fasted state, resulting in a 4.85-fold increase of the Cmax, demonstrating that the bioavailability of CBD increases with the food intake. As CBD is a highly lipophilic compound, a high-fat meal ingested at the time of dosing, which increases bile salt secretion and enhances absorption through hydrophobic barriers, may significantly increase the bioavailability of CBD, minimizing its loss. Additionally, as the formulation used in the study was not wholly pure, traces of THC may have been bioavailable. In all trials, THC, similar to the lower limit of quantification (0.125 ng/mL), was detected, demonstrating minimal interference of THC in CBD formulations.

### 3.2. Pharmacokinetic Parameters of the Oral CBD Administration in Humans with Impaired Conditions

It is crucial to establish the differences between healthy and ill patients' pharmacokinetics to establish proper treatment guidelines. For this purpose, Taylor et al. [76] studied the pharmacokinetics of a 200 mg dose administered orally in patients with mild to severe hepatic impairment versus those with normal hepatic function. The Cmax of CBD increased with the severity of hepatic impairment, compared to the control group. However, there were no significant differences in the time of maximal absorption (2.0–2.8 h) between both groups. Similarly, elimination half-times increased with the severity of hepatic impairment by 1.83-, 2.39-, and 2.57-fold in patients with mild, moderate, and severe impairment, respectively, compared to the control group. The Cmax of 7-COOH-CBD, the most abundant CBD metabolite, decreased in patients with severe hepatic impairment due to a reduced metabolic capacity for hepatic biotransformation. Furthermore, although CBD highly bonds to proteins, mainly albumin, the free drug (not bonded to proteins) was notably higher in patients with severe hepatic impairment since baseline albumin levels were lower in these patients. In contrast, renal impairment [77] had no significant effect on the pharmacokinetics of CBD. Cmax, elimination half-time, and time to maximal absorption were not affected in patients with renal impairment when compared to the control group. CBD and its primary metabolites were not detected in the urine, suggesting this likely represents a minor route of elimination of the intact drug and its derivatives. Due to increased exposure to CBD, dose reduction is highly recommended in patients with moderate to severe hepatic impairment.

### 3.3. Possible Side Effects of the CBD Oral Administration

Regarding CBD's potential side/toxic effects, different studies involving the oral administration in healthy [65–74] or mentally ill [78–87] humans have been performed to measure safety-, behavioral-, or disease-specific parameters. Results have shown good tolerance and no significant adverse effects of CBD at a wide range of dosages, from 3 to 1,200 mg/day. During the pharmacodynamic trials mentioned above [75–77], patients reported tolerance of all CBD oral administrations. The most common adverse effects reported were diarrhea, nausea, headache, and somnolence in healthy, hepatic-, and renal-impaired patients. Furthermore, there is no evidence that CBD has the potential for abuse or dependence in humans [88].

### 3.4. CBD Oral Bioavailability

CBD's oral bioavailability is poor, calculated at 8.6% compared to with the i.v. administration [89], and it is thought that 10% of the oral dose administered i.v. could exert similar effects. Due to the low oral bioavailability of CBD, modifications of the pharmaceutical formulation must be considered to increase its effectiveness in patients. These modifications are focused on overcoming poor aqueous solubility and extensive first-pass metabolism. Knaub et al. [90] used a self-emulsifying drug delivery system (SEDDS-CBD), resulting in an up to 4.4-fold increase in Cmax when compared to a commercial formulation. Similarly, Patrician et al. [91] reported an increase in Cmax with no differences in time to maximal absorption when using a modified oral CBD formulation. It is speculated that including long-chain fatty acids in the formulation could improve the uptake of CBD, allowing higher volumes of CBD to enter the circulatory system. Furthermore, different Cmax values have been observed in women and men, with greater absorption in women than men [90, 92], probably due to differences in the distribution volume, women's higher percentage of body fat on average, or hormonal status, leading to higher exposure in women in a “one-size-fits-all” dosage strategy. In a study comparing various administration routes, Bartner et al. [93] assessed the pharmacokinetics of oral CBD-infused oil, microencapsulated CBD oil beads, and CBD-infused transdermal cream. The study demonstrated that CBD-infused oil had the most favorable pharmacokinetic profile since some diffusion barriers, such as skin thickness or absorptivity, compromise transdermal absorption.

The use of CBD as a therapeutic compound is gaining popularity, and the FDA has authorized its use in the treatment of different pathologies. However, the possibility of exerting adverse effects and the uncertainty that THC may exhibit its effects due to its content in CBD formulations limits its use. Despite the high dosages administered in the aforementioned studies, the possibility of CBD causing severe adverse effects is minimal, and diarrhea, nausea, headache, and somnolence are the most commonly reported side-effects [75–77]. Unfortunately, CBD exhibits low bioavailability in oral administration, the current formulation approved by the FDA. Therefore, there is a considerable need for additional formulations with increased CBD bioavailability, as well as studies of the safety of using administration routes with increased bioavailability. Moreover, using therapeutic formulations composed of synthetic CBD could be an alternative, as no THC would be present, limiting concentration variations in the formulation, standardizing its effects, and potentially increasing its acceptance as a cardiovascular treatment.

## 4. Clinical Trials Involving CBD and the Current State of CBD as a Treatment for Ventricular Dysfunction and Heart Failure

The hallmark of cardiovascular diseases is a slow progression toward inflammatory pathologies. These are spurred by an unhealthy lifestyle, where smoking, high cholesterol, a diet rich in refined sugars, and high blood pressure lead to the endothelial activity [94]. Their clinical expressions include coronary artery disease, aortic aneurysms, peripheral artery disease, acute ischemic stroke, and venous thromboembolism [94].

Jadoon et al. [95] reported CBD's impact on blood pressure in healthy humans after an oral dose of 600 mg. In the study, the acute CBD administration reduced resting blood pressure and attenuated increases in blood pressure due to stressful situations. There are 247 clinical trials involving cannabidiol listed in the US National Library of Medicine database; most of them focus on pain relief and epilepsy, and four trials involve the measurement of cardiovascular parameters. An active study of oral dietary CBD supplements [96] in young and older healthy humans focuses on improving our understanding of how CBD might control and regulate blood vessel health, as well as cognitive and exercise performance, and includes systolic and diastolic blood pressure and heart rate measures. A completed study that assessed the effects of oral CBD (0, 200, 400, or 800 mg/Kg) on smoked marijuana's subjective, reinforcing, cognitive, and cardiovascular effects [97] evaluated the effect of CBD on the heart rate when smoking marijuana. The study showed an increase in heart rate 15 min after the inhalation of THC (5.3%/cigarette), but it decreased over time, and no improvement was found in control participants (0.01% TCH/cigarette). They found no significant effects of CBD alone on the heart rate at every concentration. Another study aims at assessing the potential cardiovascular risks and benefits of CBD therapy in children with severe epilepsy [98] by characterizing CBD's effects on EKG findings, heart rate variability, and the occurrence of seizures. The measures include Holter SDNN parameter changes, seizure frequency, and dysautonomia signs and symptoms.

Regarding heart failure, experimental and clinical evidence supports the critical role of inflammation as the central mediator in the development and progression of heart failure [99]. B-cell activation signaling, which includes proinflammatory cytokines, anticardiac antibody expression, and complement system activation, directly correlates with functional class and outcomes [100, 101]. However, as previously described, when using in vivo and ex vivo preclinical cardiovascular models, CBD has shown a substantial protective effect from fibrosis and inflammation [102, 103]. Considering the link between inflammation and heart failure, CBD emerges as a promising, alternative anti-inflammatory therapeutic approach. Currently, from total clinical protocols related to the use of CBD in several diseases, just one treats with CBD patients with heart failure A-C (CAPITAL A–C, NCT03634189) [104]. In brief, CAPITAL-AC is a phase I, prospective, open-label study designed according to SPIRIT guidelines to assess the safety of cannabidiol treatment in patients with heart failure. The sample will include 20 patients with heart failure in stages A–C (as defined by the American Heart Association/American College of Cardiology's guidelines) receiving guideline-directed medical therapy. The patients will receive cannabidiol at a maximum dose of 25 mg/kg daily with a follow-up of six months. The primary safety endpoints will be based on the incidence of adverse events. Additionally, high-sensitivity cardiac troponin T, B-type natriuretic peptide, tumor necrosis factor-*α*, and immune and collagen biomarker testing and cardiac magnetic resonance imaging will be performed at the baseline, intermediate, and final visit. CAPITAL A–C, to the best of our knowledge, is the first phase I safety trial of cannabidiol in patients with heart failure in stages A–C [104]. However, this clinical trial is not complete, and the results are not available.

Notably, in the recent SARS-COV-2 pandemic, the most frequent COVID-19 phenotype is ST-elevation myocardial infarction, including males at high risk for thromboinflammation since they have proinflammatory risk factors and cardiopulmonary comorbidities [105]. The SARS-COV-2 infection leads to thromboinflammation, endothelial cell damage, increasing platelet aggregation, and thrombi sensitivity [106, 107]. The main targets of SARS-COV-2 are endothelial cells and pericytes on the lungs and myocardium, renal, arterial, and venous vessels, including venous and arterial microcirculation. This selectivity explains the most common clinical phenotypes observed in severe COVID-19. No vaccine is available for prevention, and there is no proven effective therapy for this infection. However, extensive preclinical data indicate that CBD has significant anti-inflammatory and cardioprotective effects. CBD interacts with a range of cellular receptors, which could potentially account for its anti-inflammatory activity. Considering these protective properties, CBD could prevent cardiovascular complications and, thus, improve patient care. In this regard, a recent study that evaluated CBD's efficacy and safety in patients with COVID-19 and cardiovascular disease or risk factors was reported as a clinical trial. The study, referred to as CRDL-COVID, is a double-blind, placebo-controlled phase II–III study conducted to evaluate the effect of synthetic CBD on recovery in patients hospitalized due to COVID-19. The patients will receive cannabidiol in a maximum dose of 25 mg/kg daily with a follow-up of six months. The primary safety endpoints will be based on the incidence of adverse events. Additionally, high-sensitivity cardiac troponin T, B-type natriuretic peptide, enzyme elevation, and ECG abnormalities will be assessed as potential makers of efficacy. Then, the secondary endpoint regarding efficacy will include the percentage of patients whose COVID-19 infection requires intensive care, D-dimer elevation, the development of severe lymphopenia, and inflammatory markers. These current studies suggest that CBD is a compound with great potential to treat a variety of heart diseases. Moreover, the clinical evidence supporting its safety includes a notable lack of adverse effects. Results from the current protocol with patients who have cardiomyopathies could support the clinical use of CBD in cardiovascular diseases.

## 5. Conclusion

CBD is the most promising molecule in the family of cannabinoids, as several studies have pointed to its beneficial properties in the treatment of a wide number of diseases, including pain and anxiety control, neurodegenerative conditions, cardiovascular regulation, and perhaps, cancer. As it is derived mostly from botanical sources, a synthetic alternative is preferred because it can be obtained in a highly pure state, avoiding common contamination by undesired molecules derived from botanical purifications, such as THC, the main psychoactive compound found in cannabis [8]. CBD has been shown to have potent antioxidative effects, protecting cells from hypoxia, ischemia, and inflammatory processes. In the cardiovascular system, it can help to modulate vasorelaxation and myocardial contraction, and it is a promising therapy for chronic conditions, such heart failure, with few adverse effects or contraindications [11].

CBD has been administered to patients in a wide range of doses and found to be relatively safe. The literature suggests CBD is a compound with great potential to treat a variety of heart diseases, as animal models provide wide evidence of its cardiovascular effects, and early reports in humans evidence its low frequency of adverse effects. With the approval of different formulations of CBD by the FDA, clinical trials can be developed to ensure its efficacy in the treatment of cardiovascular diseases, including heart failure.

## Figures and Tables

**Table 1:** Effect of CBD in different cardiomyopathy experimental models.

Biological subject	CBD concentration	Experimental model	CBD treatment key results	Ref
In vitro models				
Human umbilical artery smooth muscle cells	0.1-10 *μ*M	ROS modulation with NAC	Protective effect against aberrant proliferation and migration by an increased expression of HO-1	[18]
Human aortic endothelial cells	10 *μ*M	High glucose/insulin	Decreased inflammatory (↓NF-*κβ*,) proliferation (↓JNK, ↓p70s6K), and increased survival (↑Akt) pathways	[19]
Human coronary artery endothelial cells	1.5, 3, 4.5, 6 *μ*M	High glucose-induced endothelial cell inflammatory response	Reduced mitochondrial superoxide generation, NF-*κβ* activation, and ICAM-1 and VCAM-1 expression	[20]
Primary human cardiomyocytes	4 *μ*M	Diabetic cardiomyopathy by high glucose culture	Decrease of oxidative/nitrosative stress and NF-*κβ* activation	[11]
Rat ventricular myocytes	1-10 *μ*M	Normal conditions	Inhibition of L-type Ca^2+^ channels	[22]
Cardiomyocytes(iPSC)	1 *μ*M	Ischemia/reperfusion and LPI administration	Reduced Ca^2+^ overload providing ischemia/reperfusion protection (↓GPR55 activation, ↓RhoA, ↓ROCK)	[23]
Ex vivo models				
Zucker diabetic rat aorta	10 *μ*M	Diabetic cardiomyopathy	Improved acetylcholine-induced vasorelaxation	[25]
Rat mesenteric arteries	10 mg/kg	Diabetic cardiomyopathy	Endothelium COX- and NO-dependent enhanced vasorelaxation of Ach	[26]
Human mesenteric arteries	10 *μ*M	Vasorelaxation	Promotes vasorelaxation via CB1 and the TRP activation and increased eNOS expression	[19]
Rat aorta	10 *μ*M	Contraction stress by a combination of U46619 and methoxamine	Increase vasorelaxation of precontracted aorta by inhibition of calcium channels and increased transcriptional activity of PPAR*γ*	[27]
In vivo models				
Primary and secondary hypertension rat model	10 mg/kg	Spontaneous and deoxycorticosterone acetate-salt hypertension	Reduction of cardiac and plasma oxidative stress (increased GSH and decreased GSSG) both in heart and plasma	[29]
Spontaneously hypertensive rats	3, 10 and 30 mg/kg	Hypertension	A dose-dependent decrease in HR and blood pressure mediated via TRPV1	[30]
In vivo rat I-R model	5 mg/kg	LAD ligation ischemia/reperfusion injury	A decrease in the infarct size and reduction of inflammation molecules like IL-6	[31]
In vivo I-R rabbit model	100 *μ*g/kg	Acute reperfusion myocardial infarction	Reduced infarct size and facilitated restoration of left ventricular function	[32]
In vivo rat I-R model	10, 50 *μ*g/kg	LAD ligation ischemia/reperfusion injury	Reduction of the infarct size and ventricular arrhythmias Inhibition of collagen-induced platelet aggregation	[33]
In vivo I-R rat model	50 *μ*g/kg	LAD ligation ischemia/reperfusion -induced ventricular arrhythmias	Decreased incidence and duration of ventricular tachycardia and the total length of arrhythmias by activation of the adenosine *A*_1_ receptor	[34]
Zucker diabetic rat	10 *μ*M	Diabetic cardiomyopathy	Improvement on vasorelaxation by involvement of the CB_2_ receptor and the enhancement of COX and SOD activity	[35]
Diabetic cardiomyopathy mice model	1, 10, 20 mg/kg	Streptozotocin induced diabetic cardiomyopathy	Attenuated myocardial dysfunction, cardiac fibrosis, oxidative/nitrosative stress, inflammation, and cell death	[11]
Autoimmune myocarditis mice model	10 mg/kg	MyHC*α*_334–352_ induced autoimmune myocarditis	Attenuated the CD3^+^ and CD4^+^ T cell-mediated inflammatory response and injury, and myocardial fibrosis	[5]
Doxorubicin-induced cardiomyopathy mice model	5 mg/kg	Doxorubicin-induced cardiomyopathy	Decreased serum creatine kinase-MB, cTnT, cardiac malondialdehyde, TNF-*α*, NO and Ca^2+^ levels, increased glutathione, selenium, and zinc ions levels	[36]
Doxorubicin-induced cardiomyopathy mice model	10 mg/kg	Doxorubicin-induced cardiomyopathy	Attenuated oxidative and nitrative stress, improved mitochondrial function, and biogenesis	[37]
In vivo rat stress model	1-72 mg/kg	Restraint stress	Abolished increase of HR and MAP by activation of 5-HT_1*A*_ receptor	[38–41]

**Table 2:** Use of cannabinoids in different cardiomyopathies.

Compound	Pharmacological activity	Cardiomyopathy model	Observed effect	Reference
SR144528	CB_2_ antagonist	I-R	Abolished cardioprotection of ECS, PEA, 2-AG, ACEA, and methanandamide	[50, 51, 53]
			Abolished 5-HT vasodilatory response	[52]
		Arrhythmia	No effect over ACEA protection	[58]
SR141716A (rimonabant)	CB_1_ antagonist	I-R	No effect over ECS and PEA cardioprotection	[50, 51]
			Abolished ACEA, methanandamide, and half 2-AG cardioprotection	[51, 53]
			Abolished 5-HT vasodilatory response	[52]
		MI	Prevention of hypotention followed by MI	[62]
		Arrhythmia	No effect over ACEA and HU.210 protection	[58, 59]
Anandamide (arachidinoylethilenamide, ACEA)	Endogenous cannabinoid, CB_1_ and CB_2_ agonist	I-R	Heart protection against ischemia	[51]
			Reduction of the infarct size	[53]
		Arrhythmia	Increase resistance to arrhythmogenic effects of epinephrine	[58]
			Improvement on cardiac resistance to arrhythmia	[60]
		Pulmonary artery hypertension	Endothelium-dependent pulmonary artery relaxation	[70]
2-arachidonoylglycerol (2-AG)	Endogenous cannabinoid, CB_1_ and CB_2_ agonist	I-R	Heart function recovery after reperfusion	[51]
			Protection of preconditioning on the endothelial function	[52]
Palmitoylethanolamide (PEA)	Endogenous cannabinoid, CB_1_ agonist	I-R	Heart function recovery after reperfusion	[51]
			Protection of preconditioning on the endothelial function	[52]
			No effect on infarct reduction	[53]
JWH105	CB_2_ agonist	I-R	Heart protection against ischemia	[51]
Methanandamide	Non-hydrolyzable anandamide analog	I-R	Reduction of the infarct size	[53]
		Arrhythmia	Improvement on cardiac resistance to arrhythmia	[60]
JWH-133	CB_2_ agonist	I-R	No effect on infarct reduction	[53]
			Reduction of myocardial injury	[56]
WIN55212-2	CB_2_ ligand	I-R	Protection against myocardial damage	[55]
AM630	CB_2_ antagonist	I-R	Abolished cardioprotection of WIN55212-2 and JWH-133	[55, 56]
AM251	CB_1_ antagonist	I-R	No effect on WIN55212-2 cardioprotection	[55]
HU-210	CB_1_ and CB_2_ agonist	I-R	Cardioprotection by mimic postconditioning	[57]
		Arrhythmia	Antiarrhythmic effects	[59]
Abnormal cannabidiol	Regioisomer of CBD	Diabetic cardiomyopathy	Hemodynamic, reduction of LV contractility and relaxation index	[63, 64]

## Data Availability

The data used to support the findings of this study are available from the corresponding author upon request.
